# How percentage‐protected targets can support positive biodiversity outcomes

**DOI:** 10.1111/cobi.13869

**Published:** 2022-01-17

**Authors:** Carlos Carroll, Reed F. Noss

**Affiliations:** ^1^ Klamath Center for Conservation Research Orleans California USA; ^2^ Florida Institute for Conservation Science Melrose Florida USA

**Keywords:** biodiversity target, global biodiversity framework, protected area, área protegida, marco de trabajo, global de biodiversidad, meta de biodiversidad, 保护区, 生物多样性目标, 全球生物多样性框架

## Abstract

Global targets for the percentage area of land protected, such as 30% by 2030, have gained increasing prominence, but both their scientific basis and likely effectiveness have been questioned. As with emissions‐reduction targets based on desired climate outcomes, percentage‐protected targets combine values and science by estimating the area over which conservation actions are required to help achieve desired biodiversity outcomes. Protected areas are essential for achieving many biodiversity targets, in part because many species are highly sensitive to human‐associated disturbance. However, because the contribution of protected areas to biodiversity outcomes is contingent on their location, management, governance, threats, and what occurs across the broader landscape matrix, global percentage‐protected targets are unavoidably empirical generalizations of ecological patterns and processes across diverse geographies. Percentage‐protected targets are insufficient in isolation but can complement other actions and contribute to biodiversity outcomes within a framework that balances accuracy and pragmatism in a global context characterized by imperfect biodiversity data. Ideally, percentage‐protected targets serve as anchors that strengthen comprehensive national biodiversity strategies by communicating the level of ambition necessary to reverse current trends of biodiversity loss. If such targets are to fulfill this role within the complex societal process by which both values and science impel conservation actions, conservation scientists must clearly communicate the nature of the evidence base supporting percentage‐protected targets and how protected areas can function within a broader landscape managed for sustainable coexistence between people and nature. A new paradigm for protected and conserved areas recognizes that national coordination, incentives, and monitoring should support rather than undermine diverse locally led conservation initiatives. However, the definition of a conserved area must retain a strong focus on biodiversity to remain consistent with the evidence base from which percentage‐protected targets were originally derived.

## Abstract 2

*Article impact statement*: Targets for the percentage of area under protection can advance conservation if their context and evidence base is clearly communicated.

## Abstract (es)

**Resumen:**

Las metas globales del porcentaje de área de suelo protegido, como el de 30% para el 2030, han obtenido una prominencia incrementada, a pesar de que se les cuestionen sus bases científicas y la probabilidad de su efectividad. Así como las metas de reducción de emisiones, las metas de porcentaje de protección combinan valores y ciencia mediante la estimación del área que requiere acciones de conservación para ayudar a lograr los resultados deseados de biodiversidad. Las áreas protegidas son esenciales para alcanzar muchas metas de biodiversidad, en parte porque muchas especies son altamente sensibles a las perturbaciones asociadas con el humano. Sin embargo, debido a que la contribución de las áreas protegidas a los resultados de biodiversidad depende de su ubicación, gestión, manejo, amenazas y lo que ocurra a lo largo de la amplia matriz del paisaje, las metas de porcentaje de protección son generalizaciones empíricas inevitables de los patrones y procesos ecológicos en la geografía diversa. Las metas de porcentaje de protección son insuficientes por sí solas, pero pueden complementar a otras acciones y contribuir a los resultados de biodiversidad dentro de un marco de trabajo que balancee la exactitud y el pragmatismo dentro de un contexto global caracterizado por datos imperfectos de la biodiversidad. Idealmente, las metas de porcentaje de protección fungen como pilares que fortalecen las estrategias nacionales integrales de biodiversidad mediante la comunicación del nivel de ambición necesaria para revertir las tendencias actuales de pérdida de la biodiversidad. Si se espera que dichas metas realicen este papel dentro del complejo proceso social en el que tanto los valores como la ciencia impulsan las acciones de conservación, los científicos de la conservación deben comunicar claramente la naturaleza de la base de evidencias que respalda las metas de porcentaje de protección y cómo las áreas protegidas pueden funcionar dentro de un paisaje más amplio gestionado por la coexistencia sustentable entre la naturaleza y las personas. Un nuevo paradigma para las áreas protegidas y conservadas reconoce que la coordinación nacional, los incentivos y el monitoreo deberían respaldar, y no debilitar, a las diferentes iniciativas de conservación llevadas por la población local. Sin embargo, la definición de un área conservada debe mantener un enfoque sólido sobre la biodiversidad para seguir siendo coherente con la base de evidencias de la cual derivaron originalmente las metas de porcentaje de protección.

## 保护百分比目标有利于积极的生物多样性结果

**【摘要】**关于土地保护面积百分比的全球目标(比如到2030年达到30%的土地保护面积)已经获得了越来越多的关注, 同时其科学基础和有效性也受到了质疑。与基于预期气候结果的减排目标一样, 保护百分比目标通过估计需要多大面积来采取保护行动以帮助实现预期生物多样性结果, 以来将价值观与科学相结合。保护区对于实现许多生物多样性目标来说至关重要, 一部分原因是许多物种对人类干扰十分敏感。然而, 保护区对生物多样性结果的贡献取决于其位置、管理、治理、面临的威胁以及在整个更广泛的景观基质中存在的问题, 因此, 全球保护百分比目标不可避免地是对不同地理区域的生态格局和过程的经验概括。虽然仅仅设立保护百分比目标是不够的, 但可以采取其它行动作为补充; 在生物多样性数据不完善的全球背景下, 还可以将保护百分比目标纳入平衡准确性和实用性的框架内, 以促进生物多样性保护成果。在理想情况下, 保护百分比目标可以作为锚点, 通过表现出雄心壮志来扭转当前生物多样性丧失趋势, 来强化国家生物多样性综合战略。如果想让这些目标在由价值观和科学共同推动保护行动的复杂社会过程中发挥作用, 保护科学家必须清楚地阐明支持保护百分比目标的证据基础, 以及保护区在以人与自然可持续共存为目标进行管理的更广泛的景观中如何发挥作用。新的保护区范式应认识到, 国家水平的协调、激励和监测工作应支持而非破坏地方领导的各种保护措施。然而, 在定义保护区时必须保有对生物多样性的高度关注, 以确保与最初得出保护百分比目标的证据基础保持一致。**【翻译 :胡怡思;审校 :聂永刚】**

## INTRODUCTION

Percentage targets for the area of land (or water) protected for biodiversity conservation (hereafter percentage‐protected targets) are a persistent and contentious feature of global conservation policy. To address the accelerating loss of global biodiversity (IPBES, 2019), many national governments have endorsed calls to protect at least 30% of their respective nations by 2030 (UNEP, 2020). This "30×30" commitment will likely be a key element of a new set of biodiversity targets, the Global Biodiversity Framework (GBF), to be finalized at the 15th Conference of Parties to the Convention on Biological Diversity (CBD) (CBD, 2021).

Percentage‐protected targets, such as 30×30, have grown more ambitious over time. In 1987 the Brundtland Commission suggested that the world's nations each protect at least 12% of their area (Brundtland, 1987). At its 2010 meeting, the CBD proposed that its parties (all nations except the United States and the Vatican) protect at least 17% of their terrestrial area by 2020 (CBD, 2010). With about 16% of Earth's land area now formally protected, the areal component of this goal has nearly been achieved, representing a tripling of the global protected area network within a few decades (UNEP‐WCMC, 2021).

The motivation for advancing biodiversity conservation via a global percentage‐protected target lies in the essential role protected areas play in sustaining biodiversity in the face of anthropogenic pressures and the relative feasibility of tracking increases in protected area designations (Bhola et al., 2020; Watson et al., 2014). Global percentage‐protected targets have advanced conservation, not only by directly incentivizing expansion of protected area networks, but also by helping to raise awareness of biodiversity loss, build partnerships and promote investment in conservation, and develop tools for tracking the status of biodiversity (Doherty et al., 2018; Woodley et al., 2019).

Although percentage‐protected targets appear effective in practice in part because they are simple to communicate and monitor, the difference they make to the outcome of interest—retention of diverse and resilient ecosystems that sustain human societies—is often unclear (Maron et al., 2021; Pressey et al., 2021). Some suggest that percentage‐protected targets can have unanticipated negative effects on biodiversity outcomes if national governments focus solely on areal extent of protection or implement new protected areas without regard to equity and rights of Indigenous communities (Maxwell et al., 2020; Pressey et al., 2021).

Are proposals such as 30×30 and “nature needs half” (Locke, 2014) simply a means of communicating that nature needs more? Or, do percentage‐protected targets have value beyond stimulating conservation ambition? Is it possible to retain the practical value of a simple target while improving its relevance to biodiversity outcomes? Increasing the likelihood that percentage‐protected targets contribute substantially to retention of biodiversity requires clarifying the social and ecological complexities of the linkage between protected areas and biodiversity outcomes across a diverse spectrum of land management and governance contexts.

We sought to clarify the link between percentage‐protected and biodiversity‐outcome‐based targets by drawing parallels to the analogous challenges of defining global emissions‐reduction targets and linking these targets to global climate outcomes (Table 1). We connected the evidence base supporting percentage‐protected targets with the standards necessary for defining protected and conserved areas (the latter term refers to areas managed under “other effective area‐based conservation measures” [OECM; CBD, 2018]) using a definition that is flexible but ensures that such areas contribute substantively to biodiversity outcome goals. We considered how strictly protected areas can be better integrated with landscape planning to enhance the biodiversity benefits received from areas under other types of management and how national coordination, incentives, and monitoring can support locally led conservation initiatives. Our goal in exploring these issues was to provide a nuanced understanding of the strengths and limitations of percentage‐protected targets and their value within the sociopolitical process through which conservation progress occurs.

**TABLE 1:** Stages of development and implementation process for biodiversity conservation and climate mitigation targets, and associated science and policy questions at each stage

Stages of target development and implementation process	Steps in climate change target development and implementation	Steps in biodiversity target development and implementation	Challenges in biodiversity target development and implementation process
Field observations and simulations	observations and simulations of global climate systems; quantification of observational and model‐based uncertainty	observations of impacts on biodiversity outcomes of past protected area designations; simulations of species and ecosystem response to habitat loss; systematic conservation planning	variation in protected area contribution to outcomes due to location, management, governance, threat level of protected area, and condition of landscape matrix
Empirical generalizations	summarize and generalize regional and global climate and Earth systems response to various global temperature thresholds (i.e., alternative values for climate apex target) (IPCC)	summarize above data over range of ecoregions, including via use of species‐area and other models (IPBES); describe strengths and limitations of generalizations	observational and model‐based uncertainty; multiscale nature of biodiversity and outcome targets; generalization from regional observations to global biodiversity target more difficult than with global climate systems
Negotiated choice of preferred outcome	discuss relative value and urgency of climate mitigation versus other societal goals; establish desired outcome (e.g., maximum 1.5°C or 2°C heating) (UNFCC)	discuss relative value of biodiversity versus (or as complement to) other societal goals; describe complementary nature of various targets and goals; propose and negotiate action and outcome targets in Global Biodiversity Framework (CBD)	place‐specific nature of appropriate governance model for conserved areas
Politically informed interpretation of target	determine what actions count toward nationally determined contributions (NDC), how remaining carbon budget can be fairly allocated between historical polluters and new sources, develop funding to support adaptation especially in global south; establish NDC	establish definition of areas managed under other effective conservation measures (OECM); develop national biodiversity strategies and action plans (NBSAP)	difficulty in characterizing degree to which different management categories contribute to outcomes and thus should count toward percentage target; variation in protected area resources, governance, and effectiveness
Implementation actions	establish national and subnational policies on climate mitigation; clarify respective roles of local initiative versus national policy	establish protected areas and OECM; ensure effective management and governance; overcome barriers to cross‐jurisdictional coordination	national and local coordination more complex than for climate policy
Monitoring and adaptive management	track national commitments versus actual achievements; track response of climate system; update simulations	link protected‐area‐related actions to impacts and outcomes	monitoring challenges, especially for species and intraspecific diversity; time lag between actions and biodiversity response complicate adaptive management

## HISTORY OF AND EVIDENCE BASE FOR PERCENTAGE‐PROTECTED TARGETS

Much literature addressing percentage‐protected targets tends to conflate protected area establishment (an action) and biodiversity retention (an outcome) (Maron et al., 2018). Whenever percentage‐protected targets are communicated, it is crucial that the assumptions underpinning them be clear. Is the target the required percentage of land under strict protection, such as national parks, or is it an estimate of the total area under strict protection plus multiple‐use management required to achieve desired biodiversity outcomes? In either case, is it assumed that eventually the remaining landscape will be transformed and therefore contribute little to biodiversity outcomes?

Two main lines of evidence support identification of percentage‐protected targets. First, thresholds can be directly identified in the response of biodiversity or ecosystem processes to varying levels of intact habitat or development intensity in a landscape. Odum and Odum (1972) proposed that prudent planning would retain 50% of every region as natural area to ensure maintenance of what are now called ecosystem services. Species–area relationships have also been used to justify percentage‐protected targets that would limit species extinctions to below a certain level (Wilson, 2016). Such proposals are implicitly agnostic about the extent to which the landscape outside protected areas will contribute to biodiversity or ecosystem function goals. Taken to its extreme, this might imply that all biodiversity outside protected areas will be lost (here termed the 30+0 approach).

A second prioritization‐based approach identifies sites deemed essential to capture in protected areas due to their irreplaceability or vulnerability; the total percentage of a region that is required to retain all such sites is calculated (Noss & Cooperrider, 1994). Although typically based on static conservation features, prioritizations can also include output from spatially explicit population or disturbance models (Noss et al., 2002). Global targets based on a synthesis of systematic conservation plans from many regions lead to estimates that retention of natural systems (via strict protection or other management strategies) across approximately 25–75% of an ecoregion is needed to meet well‐accepted conservation goals, such as representing all ecosystem types, maintaining viable populations of all native species, and sustaining ecological processes and resilience (Noss, 1996; Noss et al., 2012).

Many ecoregional plans also consider the contribution to conservation of lands outside strictly protected areas and what management is consistent with sustaining this contribution in combination with human economic uses. This approach in its extreme might be termed a 30+70 approach; that is, 30% is protected in a core network of protected areas embedded in a landscape managed for sustainable coexistence between humans and nature (Watson et al., 2021). Positive biodiversity outcomes often require a combination of “land sparing” (areas with limited use) and “land sharing” (areas with more intensive human use that also support biodiversity) (Kremen, 2015).

These complexities can be obscured when scientific findings are translated into simple targets, leading critics to question whether percentage‐protected targets are science based (Wilhere, 2021). Such targets may be science‐informed but unavoidably include a values‐based component arising from the instrumental value of biodiversity to humans and the proposition that biodiversity has intrinsic value and ought to be conserved (Fearnside, 2021; Noss, 1996). Percentage‐protected targets should reflect science‐based estimates of the area over which actions are required to contribute to alternative biodiversity outcomes, the latter determined based on societal values.

An analogy to the international effort to halt anthropogenic climate change is illustrative (Table 1). The IPCC synthesized the anticipated societal and ecological effects of climate change if various targets for limiting anthropogenic emissions and the consequent rise in global temperature are met (Teske, 2019). Global climate outcomes (e.g., limiting heating to 1.5 °C or 2 °C) are endorsed via values‐based societal choices that climate change effects beyond a certain level must be avoided. Then, the actions required to achieve these outcomes (a certain quantum of emissions reduction) are set based on scientific evidence.

Analogously, IPBES, an intergovernmental biodiversity science body, has sought to quantify the socioeconomic benefits from biodiversity and the costs of its loss (IPBES, 2019). As is the case with climate targets, CBD targets are informed by science but are inevitably negotiated political outcomes based on societal preferences regarding desired states of nature and tolerable risk (Table 1). Once such outcome goals are set, a science‐based process of setting percentage‐protected and other action targets occurs. Given imperfect information, such targets will be iteratively revised as new data become available.

Near‐future targets, such as 30×30, although likely inadequate over the long term, may be seen as the maximum feasible societal goal over the next decade. Many conservationists advance the proposition that the 30×30 goal is a step toward an ultimate goal of protection or retention as natural habitat of at least half of Earth (Dinerstein et al., 2019; Locke, 2014; Noss et al., 2012; Wilson, 2016;).

Even where percentage‐protected targets are framed as science‐based estimates of what is required to achieve particular outcomes, there are many reasons the estimated percent required might vary. For example, global targets are necessarily generalizations based on the diverse responses observed in multiple geographies and over multiple scales of biodiversity. Like the emissions‐reductions target required to meet the maximum global warming outcome of 2 °C, which is an inadequate target for low‐lying island nations, percentage‐protected targets that are adequate for some regions will be inadequate for others.

Research and planning at extents much smaller than global (e.g., ecoregions) are needed to determine empirically the extent of protection required to sustain biodiversity in specific geographies. Ecoregions that are more physically or biologically heterogeneous (i.e., higher beta diversity) or richer in range‐restricted species will likely require a greater percentage of area protected than more homogeneous or endemic‐poor ecoregions (Noss, 1996). Because the processes that maintain biodiversity are not globally connected to the same extent as climate systems are, better data cannot entirely resolve the inherent contrast between global percentage‐protected targets and regionally specific recommendations.

Percentage‐protected targets do not capture all factors that determine the contribution of protected areas to biodiversity outcomes (Pressey et al., 2021). The conservation impact of a protected area is contingent on its extent and location with respect to key biodiversity features; its location relative to threats that it can avert (Harfoot et al., 2021; Pressey et al., 2021); and its management and governance and their effectiveness in averting those threats. These contingencies can be addressed via additional targets and indicators that complement the percentage‐protected target (Appendix S1; UNEP‐WCMC, 2020). Because even ambitious percentage‐protected targets encompass a minority of the landscape, additional targets and indicators are essential to ensuring that protected and conserved areas are located where they can achieve the maximum impact on biodiversity outcomes (Dinerstein et al., 2019; Pressey et al., 2021).

## BETTER INTEGRATION OF PERCENTAGE‐PROTECTED TARGETS AND OUTCOME GOALS

The CBD aims to reverse biodiversity loss and safeguard nature's contributions to people in an equitable manner (CBD, 2021). Due to the hierarchical nature of biodiversity, there is no single index of the status of biodiversity akin to the IPCC's “apex target” based on global mean temperature increase (Díaz et al., 2020). The latest version of the GBF includes targets directly related to the desired outcome of halting or reversing loss of biodiversity at the ecosystem, species, and genetic levels (CBD, 2021; Díaz et al., 2020). Proposed outcome goals and milestones include net gain in extent, connectivity, and integrity of ecosystems relative to a 2020 baseline, reduction in species extinction rates and extinction risk, and retention of existing genetic diversity within species (CBD, 2021; Díaz et al., 2020; Watson et al., 2020) (Appendix S1).

Management interventions can be directly linked to biodiversity outcomes by measuring and forecasting the positive conservation impact of specific actions (Pressey et al., 2021). Estimating the additive impact of protected areas requires comparison of the outcomes expected given designation of a protected area with those expected under no protection, a function of anticipated threats and the effectiveness of a protected area in mitigating them (Pressey et al., 2021). Quantitative impact targets can guide where protected areas can be located to maximize their contribution to net gains.

Methods for assessing how varying percentages of protected area in a landscape are correlated with the extent of intact ecosystems, species extinction rate and risk, and levels of intraspecific diversity span a spectrum of complexity and ecological realism (Appendix S1). The most conceptually straightforward method of tracking the contribution of protected areas toward biodiversity outcome goals is via direct monitoring of the status of biodiversity relative to its anticipated status without protection. This is most feasible for ecosystem‐related goals. Remote sensing data can track many key attributes, such as fragmentation, that characterize intact versus degraded ecosystems and thereby determine the extent and location of new protection necessary to achieve net gain targets (Watson et al., 2020). Comprehensively monitoring the rate of species extinction and the status of intraspecific diversity is essential but more challenging (Rounsevell et al., 2020).

The 2 approaches (process based and prioritization based) described above are also relevant for tracking the contributions of protected areas to outcomes (Appendix S1). Process‐based models can be used to evaluate the adequacy of current or proposed protected area networks to achieve outcomes, in a manner analogous to modeling to assess the adequacy of proposed national climate mitigation commitments. Ecosystem modeling can project to what degree anticipated land‐use patterns or alternative management regimes will meet outcome goals related to desired ecosystem states, processes, and services. Spatially explicit population models can be used to assess the adequacy of proposed protected area networks and the larger landscape for fulfilling outcomes related to reducing extinction risk and sustaining intraspecific diversity (Pierson et al., 2015).

In a prioritization‐based approach, it can be determined whether proposed protected areas encompass sites of high conservation importance identified in systematic conservation plans (Margules & Pressey, 2000) (Appendix S1). However, most ecoregions still lack such plans, and many plans are out of date. The GBF proposes use of global data sets, such as the key biodiversity areas system, to complement information from other sources (CBD, 2021), although such global data sets are incomplete and may be biased toward well‐studied regions. The GBF also proposes augmenting species monitoring data with indirect indicators based on species distribution models (CBD, 2021). However, global species occurrence databases and suitability models derived from such data have limitations (Pressey et al., 2021).

Percentage‐protected targets can also be indirectly linked to biodiversity outcomes by monitoring newly protected areas in terms of their representativeness, based on data or models of the distribution of ecosystems and species, and connectivity, based on structural connectivity metrics (CBD, 2021). Although these metrics are available as globally consistent data sets, they do not directly track ecological processes of interest. For example, the connectivity metrics included in the GBF are abstracted representations of functional population connectivity in real landscapes (Schumaker et al., 2014). Caution is also necessary in using coarse‐scale units, such as ecoregions, to assess representation due to their high levels of internal ecological heterogeneity (Pressey et al., 2021) (Appendix S1).

Given that the GBF will be applied globally, the data requirements associated with direct monitoring of species and populations, process‐based models, and systematic conservation planning are formidable. Conversely, globally consistent indicators (e.g., as derived from remotely sensed data) often have limited spatial and thematic resolution (Pressey et al., 2021). Given the strengths and limitations of each of these approaches, the GBF appropriately envisions use of a combination of methodologies to monitor progress and retains complementary action and outcome targets in an effort to balance accuracy and pragmatism in a global context characterized by imperfect biodiversity data.

## BETTER INTEGRATION OF CONSERVED AREAS INTO LANDSCAPES

A potentially larger source of uncertainty in linking percentage‐protected targets and outcome goals involves what happens in the broader landscape (i.e., whether strictly protected areas will be surrounded by developed matrix or instead complemented by other conservation areas and sustainable resource management) (Maxwell et al., 2020; Watson et al., 2021). That protected areas should go hand‐in‐hand with conservation across the broader landscape is well accepted and formed the impetus for the Man and the Biosphere (MAB) program's concept of biosphere reserves (Batisse, 1982) and other conservation zoning approaches, such as multiple‐use modules (Noss & Harris, 1986).

Such planning approaches situate core protected areas in a matrix of buffer and transition zones and other lands used for sustainable resource production (Noss & Harris, 1986). The strictest level of protection is appropriate for sites that are more irreplaceable or vulnerable, with a gradient of decreasing protection that parallels gradients in decreasing irreplaceability. The outcome of biodiversity retention is achieved by a combination of strictly protected areas and other management mechanisms that retain nature throughout the landscape (i.e., the 30+70 approach), in which landscape‐wide planning complements designation of protected areas to achieve desired outcomes.

The GBF recognizes that protected areas function in the context of landscapes and supports management of entire landscapes for sustainable coexistence between people and nature (CBD, 2021). The condition of a landscape is fundamental in fulfilling certain targets, such as maintaining adequate connectivity between protected areas and allowing ecological processes that operate on large spatial scales to continue functioning. However, this recognition is not always retained when global percentage‐protected targets are implemented at national extents. For example, initial statements from the U.S. federal 30×30 initiative emphasize landscape‐scale planning (e.g., enhanced focus on biodiversity on multiple‐use public lands plus incentivizing such focus on private lands) as an alternative to protected area designation (Yachnin, 2021), despite the substantial research indicating that protected areas are elements of landscape‐level planning that are essential for achieving many biodiversity outcomes, in part because many species cannot persist even at relatively low levels of human disturbance (Pacifici et al., 2020; Watson et al., 2014).

## A DEFINITION OF *CONSERVED AREA* THAT SUPPORTS POSITIVE BIODIVERSITY OUTCOMES

Early percentage‐protected targets implicitly referenced the traditional model of a protected area established and managed by a central government. Beginning with the CBD's 2010 17% target, the land management categories that counted toward the target were expanded to include OECM (CBD, 2010). This shift was motivated by concerns that the standard park model was inappropriate in certain sociopolitical contexts (Jonas et al., 2021). The OECM standards, developed by international organizations, including CBD and International Union for the Conservation of Nature (IUCN), focus on whether an area provides positive and sustained benefits to biodiversity and has a management plan that explicitly provides for these benefits (CBD, 2018). Based on the IUCN definition, such areas have 4 necessary components: good governance, sound design and planning, effective management, and successful conservation outcomes (Hockings et al., 2019).

Given the incentive for national governments to report substantial (and perhaps inflated) progress toward percentage‐protected targets, it is challenging to define OECM in a flexible yet substantive manner. This is analogous to ensuring that nationally determined contributions (NDC) and national climate policies align with the emissions‐reduction targets endorsed by parties to global agreements (Liu & Raftery, 2021). National governments can distort implementation of percentage‐protected targets by siting conservation areas without regard to the distribution of biodiversity (e.g., on lands with low economic value) or by counting areas toward the target even though their existing land use is incompatible with biodiversity outcomes. When a metric becomes a target, it ceases to be an accurate metric (Goodhart's law) because it can be manipulated (i.e., disconnected from biodiversity outcomes) (Newton, 2011). Effective target implementation may require a rigorous global system to track achievement, similar to that used to track achievement of NDCs (Table 1) (Xu et al., 2021). Determining what land uses are compatible with a conserved area hinges on the issue of defining thresholds along a continuum of biodiversity response to varying types and intensities of land use and management. Terrestrial conservation planners can learn from existing frameworks developed to classify marine reserves along a gradient from fully to minimally protected (Grorud‐Colvert et al., 2021).

Integration of action targets and outcome goals can be furthered by an effective definition of *conserved area* (OECM) or could be hindered by a definition that lacks a substantive connection to biodiversity outcomes (Simmons et al., 2021). If OECMs are to count toward percentage‐proposed targets, this will require a strong focus on biodiversity in the definition of OECM to remain consistent with the evidence base from which the percentage‐protected target was originally derived (Noss et al., 2012). Effects of human‐associated disturbance on biodiversity are a function of disturbance area and intensity (Suraci et al., 2021). The core questions are what pattern and intensity of human land use is compatible with desired biodiversity outcomes and how this pattern and intensity can be achieved equitably. Potentially, to achieve an outcome equivalent to that achieved by strictly protecting 30% of the landscape, planners could conserve a proportion >30% under an OECM definition that allows a greater range of land uses but still explicitly focuses on biodiversity. The validity of this approach depends on the extent to which the conservation features of concern depend on strictly protected areas, which may require maintaining a minimum percentage under such protection (Pacifici et al., 2020).

The Gap Analysis Program's (GAP) Protected Status categories are often used to estimate the total protected area in the United States (Scott et al., 1993). Categories 1 and 2 are typically strictly protected areas. Most U.S. public lands are category 3 (i.e., they are managed for multiple uses but protect federally listed species and do not result in permanent land conversion) (Scott et al., 1993). In practice, however, different GAP3 lands experience widely varying land management regimes and therefore show contrasting levels of intactness and contributions to biodiversity.

A workable OECM definition needs to distinguish GAP3 lands for which the sum effects of all existing land uses and management actions in an area substantially contribute to positive biodiversity outcomes from those that do not, for example, due to ongoing uses that contribute to degradation (Maron et al., 2020). Given the overarching goal of sustaining biodiversity, any definition of land uses compatible with OECM must respect the precautionary principle embodied in OECM standards by placing the burden on managers and policy makers to demonstrate compatibility of land management with positive biodiversity outcomes (CBD, 2018, 2021). A comprehensive OECM standard needs to address biodiversity status and trends in a particular area. Does ongoing restoration of degraded lands place them in the OECM category despite a modest current ability to sustain biodiversity? A key question is whether inclusion of such areas in the OECM category enhances or compromises adequacy of the percentage‐protected target.

## A NEW SOCIETAL PARADIGM FOR CONSERVED AREAS

Much of the impetus for development of the OECM concept originated from critiques of the existing paradigm for establishment of protected areas (Jonas et al., 2021; Maxwell et al., 2020). What we have termed the 30+0 perspective (i.e., assumption that strictly protected areas are the primary strategy for biodiversity retention) arises in part from the reality of conservation in rapidly developing landscapes, where the landscape matrix is being radically transformed with consequent loss of ability to support many native species (Terborgh, 2020). However, this approach has been criticized as a “fortress conservation” strategy that, if interpreted as a landscape rigidly divided between areas for people and for wildlife, can result in eviction and loss of rights of Indigenous and local communities (Brockington, 2002).

Examples exist of Indigenous dispossession during protected area establishment worldwide. However, in other contexts, establishment of Indigenous‐managed protected areas has served as an effective defense for Indigenous communities fighting dispossession by local elites and global extractive industries. For example, Indigenous organizations recently secured support from IUCN for a proposal to protect 80% of the Amazon basin (IUCN, 2021). The global extent of Indigenous cultural landscapes (Fletcher et al., 2021) demonstrates that the concept of wilderness must encompass areas that support subsistence and management practices of Indigenous communities while also supporting the full complement of native species and ecological processes that sustain biodiversity over evolutionary time scales (Watson & Venter, 2021).

Efforts to overcome historical limitations of the protected area concept have led to development of a new paradigm for protected and conserved areas in which national coordination, incentives, and monitoring support rather than usurp control from Indigenous and local community conservation initiatives (Jonas et al., 2021). This new paradigm recognizes that equitable and effective governance and effective management, reporting, and monitoring are preconditions for positive conservation outcomes in protected areas.

An example of the new paradigm is the Canadian federal government's commitment to meeting CBD targets through reconciliation with First Nations within regional land‐use planning processes and creation of Indigenous protected and conserved areas (IPCA) (Tran et al., 2020). The land‐use plan for the Peel Watershed, Yukon, ratified in 2019 as part of this process, confers some level of protected status on 83% of the watershed. Planning was led by First Nations and subnational governments, and federal support complemented local planning processes that integrated Western science and traditional ecological knowledge (PWPC, 2019).

Many questions remain about how to achieve effective biodiversity outcomes within the context of the new protected areas paradigm. What do goals of equity and respect for land rights imply in a context where local sentiment about land conservation is polarized? Representative democracy does not assume the public has a unified perspective; rather, it provides a framework for acting in the face of diverse perspectives. For example, to meet commitments for reducing emissions, national governments have created alternative employment opportunities in coal mining regions rather than protect existing mining jobs.

To sustain biodiversity, ecocentric values and objectives (Taylor et al., 2020) may need to similarly take precedence over potential short‐term economic opportunities. Designation of the Bear Ears National Monument in the western United States, an area encompassing Indigenous cultural landscapes and sought‐after mineral deposits, provides an example in which the national government privileged the rights of Indigenous residents over those economically tied to extractive industries (Creadon & Bergren, 2019). To an even greater extent than in the case of climate policy, the best governance process will be place specific and require transformational change in societal structures (Grumbine & Xu, 2021; No'kmaq et al, 2021). Successful implementation of the GBF will require financial support from the Global North for conserved areas in less‐developed nations, analogous to the role of the Paris Agreement's Green Climate Fund (UNFCCC, 2015).

## CONCLUSIONS

The transformative change required to respond effectively to the biodiversity and climate crises is a complex societal process through which values and science impel targets and resultant actions (Grumbine & Xu, 2021; No'kmaq et al, 2021). The debate over biodiversity targets is in many ways analogous to the debate over the degree of ambition necessary to limit global heating. Action targets, such as 30×30, are a necessary complement to biodiversity outcome goals because they play a fundamental role in informing the societal process by which national conservation goals are proposed and implemented.

Nevertheless, the societal debate concerning the appropriate level of conservation ambition should not obscure scientific understanding of the complex relationship between conservation actions and biodiversity outcomes. The contribution of protected areas to biodiversity outcomes is contingent on their location, management, governance, existing threats, and what occurs in the broader landscape. Percentage‐protected targets are unavoidably empirical generalizations, which are insufficient in isolation but can be evidence based if applied as part of a comprehensive suite of targets, such as the proposed GBF (CBD, 2021). Achievement of percentage‐protected targets must not overshadow the overarching biodiversity outcomes to which the target is meant to contribute. The primary focus must remain on the outcomes of net gain in biodiversity at all scales and levels of organization, recognizing that the sustainability of society requires a functioning biosphere as a requirement for all life (Locke et al., 2021).

Global targets need to be supplemented by ecoregion‐specific conservation plans that determine how much is enough in each ecoregion to achieve conservation goals. What is possible to achieve for conservation in an ecoregion with abundant remaining wild area is quite different from what can be achieved in an ecoregion dominated by intensive agricultural or urban land uses. Conversely, substantial restoration may be required in highly depleted ecoregions if they are to sustain their existing biodiversity, due to time lags in biodiversity response to land‐cover change (Maron et al., 2021). Until such ecoregional plans become available, it is appropriate to proceed on the basis of the best available information, including empirical generalizations (Noss et al., 2012).

If percentage‐protected targets, such as 30×30, are implemented within the context of broader frameworks, such as the GBF, they can serve as anchors of comprehensive national biodiversity strategies and help communicate the level of ambition necessary to reverse current trends of biodiversity loss via a variety of existing and new conservation policies. As with the climate crisis, there is a need to encourage individual and local actions in response to the biodiversity crisis while recognizing that the enormity of the challenge requires ambitious, coordinated national efforts that complement local efforts.

## Supporting information

Supporting Information Table S1. Data and model‐based approaches for monitoring achievement of biodiversity outcome goals in relation to percentage‐protected targets.
