# Supplementary material for: How percentage‐protected targets can support positive biodiversity outcomes
Source: Conserv Biol. 2022 Jan 17;36(4):e13869. doi: 10.1111/cobi.13869 (PMC9540251; doi:10.1111/cobi.13869)
Supplement: Supplementary file 1 — Supporting Information Table S1. Data and model‐based approaches for monitoring achievement of biodiversity outcome goals in relation to percentage‐protected targets. [file COBI-36-0-s001.pdf]

Supporting Information for 'How percentage-protected targets can support positive biodiversity outcomes'.

Carlos Carroll[1], Reed F. Noss[2].

1. Klamath Center for Conservation Research, Orleans, CA 95556 USA.

2. Florida Institute for Conservation Science, Melrose, FL 32666 USA.

Supporting Information Table S1. Data and model-based approaches for monitoring achievement of biodiversity outcome goals in relation to percentage-protected targets. See UNEP-WCMC website "Indicators for the post-2020 global biodiversity framework" (https://www.post-2020indicators.org/) for further information on several of these metrics and others not listed here.

**Scale of biodiversity**	**Data source**	**Model**		**Outcome targets by scale of biodiversity**	**Indicators relevant to percentage-protected targets**	**Challenges and limitations**
1. Ecosystem	Remote sensing of land and climate attributes	Ecosystem classification[1], intactness[2], connectivity[3], ecosystem process[4] (e.g., disturbance)		Net gain in area of intact ecosystems	Representation of conservation features within ecoregions[5], intactness	Heterogeneity within ecoregions, limitations on remotely sensed indicators of ecosystem intactness, ecosystem classification issues
2. Species	a. Environmental attributes from remote sensing, ground observations, climate projections, etc.)	Species distribution models (SDM)[6]	Red List and other expert-based thresholds[7], species-area relationship[8], spatially-explicit population models[9]	Reduction in species extinction rate and risk	Hotspots of species richness and endemism, Species Protection Index[10]	Limitations on explanatory power of SDMs and species-area models, limitations in expert-based extinction risk thresholds
	b. Monitoring data on species abundance and distribution		Red List and other expert-based thresholds[11]	Reduction in species extinction rate and risk	Representation of imperiled species[12]	Limited availability of monitoring data
3. Intraspecific diversity	Monitoring of species distribution, genetic variation	Ecotype classification[13]		Retention of existing diversity	Representation of ecotypes or other divisions within ranges[14]	Limited correspondence between environmental attributes and genetic types

4. Multi-scale site-based	KBA database[15], species distribution data or models	Systematic conservation planning[16], representation (gap) analysis[17]	Multi-scale: No loss of irreplaceable sites	Representation of irreplaceable sites, KBA[18]	KBA database limitations, inconsistencies among SCP processes

Footnotes. 1. Watson et al., 2013; 2. Watson et al., 2018; 3. Saura et al., 2018; Ward et al., 2020; 4. Leroux et al., 2007; 5. Dinerstein et al., 2017; 6. Warren et al., 2018; 7. Allan et al., 2019; 8. Wilson, 20169. Carroll et al., 2006; 10. GEOBON, 2015; 11. Rodrigues et al., 2006; 12. Faith et al., 2008; 13. Hanson et al., 2020; 14. Rochat et al., 2021; 15. Eken et al., 2004; 16. Margules & Pressey, 2000; 17. Rodrigues et al., 2004; 18. Allan et al., 2019; Dinerstein et al., 2020.

REFERENCES

Allan, J. R., Possingham, H. P., Atkinson, S. C., Waldron, A., Di Marco, M., Adams, V. M., Butchart, S. H. M., Venter, O., Maron, M., Williams, B. A., Jones, K. R., Visconti, P., Wintle, B. A., Reside, A. E., & Watson, J. E. M. (2019). Conservation attention necessary across at least 44% of Earth's terrestrial area to safeguard biodiversity. *bioRxiv*, 839977. https://doi.org/10.1101/839977

Carroll, C., Phillips, M. K., Lopez-Gonzalez, C. A., & Schumaker, N. H. (2006). Defining Recovery Goals and Strategies for Endangered Species: The Wolf as a Case Study. *BioScience*, *56*(1), 25-37. https://doi.org/10.1641/0006-3568(2006)056[0025:Drgasf]2.0.Co;2

Dinerstein, E., Joshi, A. R., Vynne, C., Lee, A. T. L., Pharand-Deschênes, F., França, M., Fernando, S., Birch, T., Burkart, K., Asner, G. P., & Olson, D. (2020). A "Global Safety Net" to reverse biodiversity loss and stabilize Earth's climate. *Science Advances*, *6*(36), eabb2824. https://doi.org/10.1126/sciadv.abb2824

Dinerstein, E., Olson, D., Joshi, A., Vynne, C., Burgess, N. D., Wikramanayake, E., Hahn, N., Palminteri, S., Hedao, P., Noss, R., Hansen, M., Locke, H., Ellis, E. C., Jones, B., Barber, C. V., Hayes, R., Kormos, C., Martin, V., Crist, E., Sechrest, W., Price, L., Baillie, J. E. M., Weeden, D., Suckling, K., Davis, C., Sizer, N., Moore, R., Thau, D., Birch, T., Potapov, P., Turubanova, S., Tyukavina, A., de Souza, N., Pintea, L., Brito, J. C., Llewellyn, O. A., Miller, A. G., Patzelt, A., Ghazanfar, S. A., Timberlake, J., Klöser, H., Shennan-Farpón, Y., Kindt, R., Lillesø, J.-P. B., van Breugel, P., Graudal, L., Voge, M., Al-Shammari, K. F., & Saleem, M. (2017). An Ecoregion-Based Approach to Protecting Half the Terrestrial Realm. *BioScience*, *67*(6), 534-545. https://doi.org/10.1093/biosci/bix014

Eken, G., Bennun, L., Brooks, T. M., Darwall, W., Fishpool, L. D. C., Foster, M., Knox, D., Langhammer, P., Matiku, P., Radford, E., Salaman, P., Sechrest, W., Smith, M. L., Spector, S., & Tordoff, A. (2004). Key Biodiversity Areas as Site Conservation Targets. *BioScience*, *54*(12), 1110-1118. https://doi.org/10.1641/0006-3568(2004)054[1110:Kbaasc]2.0.Co;2

Faith, D. P., Ferrier, S., & Williams, K. J. (2008). Getting biodiversity intactness indices right: ensuring that 'biodiversity' reflects 'diversity'. *Global Change Biology*, *14*(2), 207-217. https://doi.org/https://doi.org/10.1111/j.1365-2486.2007.01500.x

GEOBON. (2015). Global Biodiversity Change Indicators. Version 1.2. *Leipzig: Group on Earth Observations Biodiversity Observation Network Secretariat*.

Hanson, J. O., Rhodes, J. R., Butchart, S. H. M., Buchanan, G. M., Rondinini, C., Ficetola, G. F., & Fuller, R. A. (2020). Global conservation of species' niches. *Nature*, *580*(7802), 232-234. https://doi.org/10.1038/s41586-020-2138-7

Leroux, S. J., Schmiegelow, F. K. A., Lessard, R. B., & Cumming, S. G. (2007). Minimum dynamic reserves: A framework for determining reserve size in ecosystems structured by large disturbances. *Biological Conservation*, *138*(3), 464-473. https://doi.org/https://doi.org/10.1016/j.biocon.2007.05.012

Margules, C. R., & Pressey, R. L. (2000). Systematic conservation planning [10.1038/35012251]. *Nature*, *405*(6783), 243-253. http://dx.doi.org/10.1038/35012251

Rochat, E., Selmoni, O., & Joost, S. (2021). Spatial areas of genotype probability: Predicting the spatial distribution of adaptive genetic variants under future climatic conditions. *Diversity and Distributions*, *n/a*(n/a). https://doi.org/https://doi.org/10.1111/ddi.13256

Rodrigues, A. S., Pilgrim, J. D., Lamoreux, J. F., Hoffmann, M., & Brooks, T. M. (2006). The value of the IUCN Red List for conservation. *Trends Ecol Evol*, *21*(2), 71-76.

Rodrigues, A. S. L., Akçakaya, H. R., Andelman, S. J., Bakarr, M. I., Boitani, L., Brooks, T. M., Chanson, J. S., Fishpool, L. D. C., Da Fonseca, G. A. B., Gaston, K. J., Hoffmann, M., Marquet, P. A., Pilgrim, J. D., Pressey, R. L., Schipper, J., Sechrest, W., Stuart, S. N., Underhill, L. G., Waller, R. W., Watts, M. E. J., & Yan, X. (2004). Global Gap Analysis: Priority Regions for Expanding the Global Protected-Area Network. *BioScience*, *54*(12), 1092-1100. https://doi.org/10.1641/0006-3568(2004)054[1092:Ggaprf]2.0.Co;2

Saura, S., Bertzky, B., Bastin, L., Battistella, L., Mandrici, A., & Dubois, G. (2018). Protected area connectivity: Shortfalls in global targets and country-level priorities. *Biological Conservation*, *219*, 53-67. https://doi.org/https://doi.org/10.1016/j.biocon.2017.12.020

Ward, M., Saura, S., Williams, B., Ramírez-Delgado, J. P., Arafeh-Dalmau, N., Allan, J. R., Venter, O., Dubois, G., & Watson, J. E. M. (2020). Just ten percent of the global terrestrial protected area network is structurally connected via intact land. Nat Commun, 11(1), 4563.

Warren, R., Price, J., Graham, E., Forstenhaeusler, N., & VanDerWal, J. (2018). The projected effect on insects, vertebrates, and plants of limiting global warming to 1.5°C rather than 2°C. *Science*, *360*(6390), 791-795. https://doi.org/10.1126/science.aar3646

Watson, J. E. M., Evans, T., Venter, O., Williams, B., Tulloch, A., Stewart, C., Thompson, I., Ray, J. C., Murray, K., Salazar, A., McAlpine, C., Potapov, P., Walston, J., Robinson, J. G., Painter, M., Wilkie, D., Filardi, C., Laurance, W. F., Houghton, R. A., Maxwell, S., Grantham, H., Samper, C., Wang, S., Laestadius, L., Runting, R. K., Silva-Chávez, G. A., Ervin, J., & Lindenmayer, D. (2018). The exceptional value of intact forest ecosystems. *Nature Ecology & Evolution*, *2*(4), 599-610. https://doi.org/10.1038/s41559-018-0490-x

Watson, J. E. M., Iwamura, T., & Butt, N. (2013). Mapping vulnerability and conservation adaptation strategies under climate change. *Nature Climate Change*, *3*(11), 989-994. https://doi.org/10.1038/nclimate2007

Wilson, E. O. (2016). *Half-earth: our planet's fight for life*. WW Norton & Company.
